# Metals and Microbes: Microbial Community Diversity and Antibiotic Resistance in the Animas River Watershed, Colorado, USA

**DOI:** 10.3390/microorganisms14010222

**Published:** 2026-01-18

**Authors:** Jennifer L. Lowell, Lucas Brown

**Affiliations:** School of Arts and Sciences, Department of Public Health, Fort Lewis College, Durango, CO 81301, USA

**Keywords:** microbial diversity, antibiotic resistance, metals

## Abstract

Antimicrobial resistant (AMR) infections are a persistent public health issue causing excess death and economic impacts globally. Because AMR in clinical settings is often acquired from nonpathogenic bacteria that surround us, environmental surveillance must be better characterized. It has been well established that metals can co-select for bacterial AMR. Furthermore, recent studies have shown that compromised microbial community diversity may lead to community invasion by antibiotic resistance genes (ARGs). Widespread legacy mining has led to acid mine drainage and metal contamination of waterways and sediments throughout the western United States, potentially compromising microbial community diversity while simultaneously selecting for AMR bacteria. Our study objectives were to survey metal contaminated sediments from the Bonita Peak Mining District (BPMD) in southwestern Colorado, USA, compared to sites downstream in Durango, CO for bacterial and ARG diversity. Sediment bacteria were characterized using 16S rRNA Ilumina and metagenomic sequencing. We found that overall, bacterial diversity was lower in metal-contaminated, acidic sites (*p* = 0.04). Metagenomic sequencing revealed 31 different ARGs, with those encoding for efflux pumps (*mex* and *spe* gene families) substantially more prevalent in the BPMD sites, elucidating a specific AMR marker fingerprint from the high metal concentration sediments. Raising awareness and providing antimicrobial tracking techniques to resource limited communities could help provide information needed for better antibiotic use recommendations and environmental monitoring.

## 1. Introduction

Antimicrobial resistance (AMR) has been recognized by leading health agencies, including the World Health Organization, the European Centre for Disease Prevention and Control, and the Centers for Disease Control and Prevention, for decades [1,2,3]. Despite this recognition, nearly 3 million people in the United States are affected each year by antibiotic resistant bacterial infections, leading to around 35,000 deaths and costing the U.S. healthcare system over USD 4 billion [2]. Globally, it is predicted that the annual number of deaths will increase to 10 million by 2050 if effective solutions are not found to slow increasing antibiotic resistance trends [3,4]. This challenge is exacerbated by the fact that many antibiotic resistant pathogens are descended from or acquire antibiotic resistance genes (ARGs) from environmentally ubiquitous bacteria that surround us [5,6,7]. However, most innocuous environmental bacteria that may harbor ARGs or release them into the environment cannot be cultured [8] complicating assessment and tracking methods [9]. The widespread distribution of AMR bacteria and ARGs in water, sediments, and soils has led some researchers to suggest classifying them as emerging environmental contaminants requiring formalized risk assessments and integrated management strategies [7,10,11,12,13,14]. Despite this, routine surveillance remains primarily a clinical endeavor [15,16]. This disconnect is critical because environments under anthropogenic impacts may suffer altered bacterial diversity, making them more susceptible to the invasion and establishment of AMR bacteria and ARGs. Indeed, studies show that highly diverse bacterial communities, such as those observed by [17], tended to harbor lower abundance and less diverse populations of ARGs, suggesting that high diversity may serve as a protective barrier against ARG immigration. Others have shown that the resistome is specific to gut and environmental microbiome community membership [18]. Furthermore, structured environments where long-term, diversity-based resilience can evolve may provide additional protection against ARG dissemination. Conversely, certain pollutants, especially metals, which diminish microbiome diversity and co-select for antibiotic resistance, may have a greater potential to maintain ARG reservoirs even in the absence of antibiotics’ selective pressure [14]. Co-selective mechanisms including co- or cross-resistance or co-regulation of metal resistance pathways arise when resistance genes are located on the same genetic element. Efflux pump systems that eliminate metals work congruently to eliminate antibiotics from the cell [14]. This is especially relevant in aquatic environments where stream sediments are a major site for metal deposition. As a result, sediment biofilms are exposed to higher metal concentrations than those found in other microhabitats. Anthropomorphic metal inputs to streams contribute to diminishing bacterial diversity [19,20,21,22] and could potentially increase ARG abundance and diversity through invasion and co-selection [14,17,23].

Such environmental activities that lead to elevated metals in streams are intrinsically linked to societal impacts, which are varied and range from increased economic opportunities to detrimental environmental and human health outcomes. A main contributor of metals to aquatic environments is mining. While mine waste handling has improved drastically over the last century, legacy mining has left behind widespread non-point source contamination that continues to leach metals into streams throughout the western U.S. [24,25]. Decisions regarding clean-up efforts focus on environmental and human health risk assessments without incorporating microbial considerations [25]. Furthermore, communities affected by mining and the resultant environmental contamination are overwhelmingly rural and lack the resources and specialized expertise required for current methods that examine microbial communities or the antibiotic resistome. Rural communities are also not as equipped as urban communities to address bacterial infections or AMR. Typically, access to healthcare is limited, public health agencies are understaffed or underprepared, environmental health professionals have limited training in infectious disease control and prevention, antimicrobial stewardship programs in clinical settings are lacking, and access to large research institutions is limited [26,27]. Although antimicrobial stewardship programs in rural hospitals have improved over the last ten years, in Colorado, USA, 51% percent of rural infection control professionals (ICPs) reported no access to infectious disease physicians (compared to 0.0% urban ICPs) and 81.8% of rural hospitals reported no antimicrobial stewardship programs [28,29]. Furthermore, southwestern CO is designated as a Health Provider Shortage Area (HPSA). Some populations of the southwestern U.S. experience higher morbidity and mortality from infectious diseases including MRSA and drug-resistant pneumonias compared to the general U.S. population [30]. Bacterial isolates collected from western CO including MRSA, *Enterococcus* spp., *Klebsiella* spp., *Enterobacter* spp., and *Pseudomonas* show intrinsic resistance to multiple frontline antibiotics including Penicillin, Amoxicillin, and Trimethoprim [31]. Most of these genera emerged from soil and water to become clinically important [5,6,7,32]. As more pathogens become antibiotic resistant, treatment options will continue to become more complex, more expensive, and less accessible, especially for rural and socioeconomically depressed populations [27]. Therefore, selection mechanisms for AMR and the general distribution of ARGs in environments surrounding rural low resourced communities deserve to be better understood. The objectives of this study were to investigate the following from rural southwestern CO: (1) to characterize microbial communities in stream sediments impacted by legacy mining compared to downstream less impacted sites, (2) to survey a subset of sites for ARG diversity and AMR markers, and (3) to compare metal concentrations, microbial community diversity, and ARG diversity and abundance. To our knowledge, this is the first study that examines microbial community diversity in a mountainous rural area of southwestern CO that has been under the influence of mining for over 100 years, while providing a survey of associated ARGs and AMR markers.

## 2. Materials and Methods

### 2.1. Sites and Sediment Sampling and Processing

**Figure 1:**
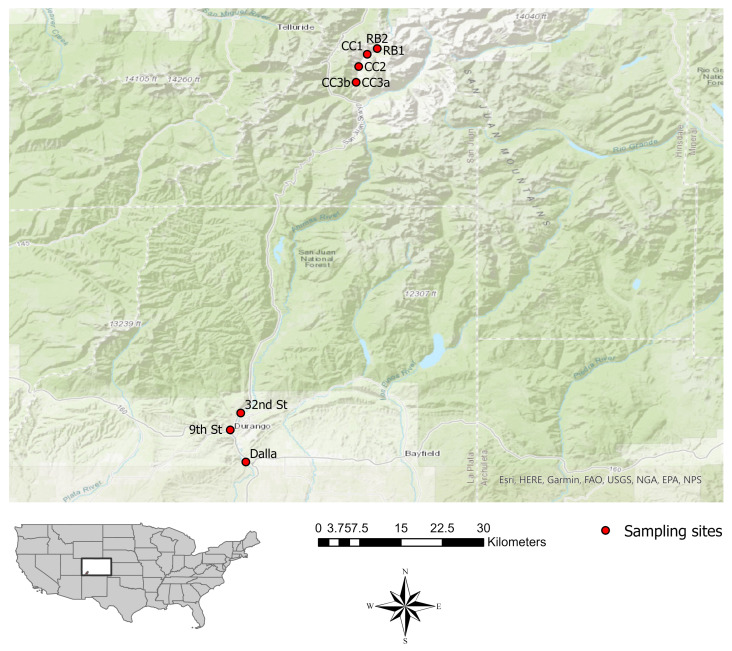
Map depicting sampling sites. The white rectangle in the U.S. map represents the location of Colorado, USA. Sampling sites are located in the southwest corner of CO in San Juan and La Plata counties depicted by the marked area. All sampling sites within the BPMD lie to the to the north, while the Durango town sites lie to the south. RB1 and RB2 are represented by overlapping points as they are approximately 20 m apart, as are CC3a and CC3b.

The Bonita Peak Mining District (BPMD) located in the San Juan Mountains of southwestern CO, lies within the confines of the Animas River Watershed. As a designated Superfund site the BPMD is characterized by historic and ongoing releases from mining operations in the Mineral Creek, Cement Creek, and the Upper Animas drainages, which converge into the Animas River near Silverton, CO, USA [25]. The Animas River flows south through Durango, CO, the Southern Ute Indian Reservation, and then to New Mexico, USA, where it meets the San Juan River. Downstream communities are dependent on these waters for drinking, irrigation, livestock, and recreation. Sediment samples were collected from six BPMD locations including two sites below the Red and Bonita mine adit discharge (RB1 and RB2), and four sites transecting Cement Creek leading into Silverton, (CC1, CC2, CC3a, and CC3b). Three sites downstream in Durango, CO, which are popular for recreation included the 32nd St Animas River access (32nd St), the 9th St Animas River access (9th St), and Dallabetta Park (Dalla) (Figure 1). Sediment was collected from each site in three replicates, at a depth of approximately 5 cm. Samples were placed in sterile tubes and transported on ice. Temperature, dissolved oxygen, pH, and total dissolved solids (turbidity) were also measured using a YSI Pro Plus multimeter (Xylem Inc., Washington, D.C., USA). Following sample transport, replicates were frozen at −80° C for molecular analyses. A fourth sediment sample was collected from each site, sent to Green Analytics Laboratory (Durango, CO), and tested for total Al, Fe, Mg, As, Cu, Pb, and Zn. Total Al, Fe, and Mg were analyzed using Inductively Coupled Plasma (ICP) according to the Environmental Protection Agency (EPA) method 6010B. Total As, Cd, Cu, Pb, and Zn were analyzed via Inductively Coupled Plasma Mass Spectrometry (ICP-MS) according to EPA method 6020A.

### 2.2. DNA Extractions and 16S rRNA Sequencing

Total genomic DNA was extracted from 0.5 gm sediment using the DNeasy PowerSoil Pro Kit (Cat. No. 47014), QIAGEN, Inc. (Germantown, MD, USA) according to the manufacturer’s protocol. One extraction blank was run per batch of extractions to confirm the absence of DNA contamination. The quality and quantity of the extracted DNA was assessed using a NanoDrop™ (Thermo Fisher, Carlsbad, CA, USA).

Extracted DNA was sent to the Center for Microbial Exploration at the University of Colorado, Boulder, CO, USA. Polymerase chain reaction was performed in duplicate using Platinum II Hot Start Master Mix (Thermo Fisher) and 515F/806R primers as described by the Earth Microbiome Project [33,34]. No template controls were processed with the samples. After confirming amplification via gel electrophoresis, amplicons were cleaned and normalized using the SequalPrep Normalization Kit (Thermo Fisher). Pooled libraries were quantified using both the Qubit dsDNA High Sensitivity Assay (Thermo Fisher) and the KAPA Library Quantification Kit for Illumina platform (Roche, Pleasanton, CA, USA). The 16S library was sequenced on a MiSeq instrument using a v2 300-cycle kit (Roche) with a 15% phiX spike.

### 2.3. Microbial Community and Statistical Analysis

Sequences were analyzed with Quantitative Insights Into Microbial Ecology 2 (QIIME2, version 2024.10) software [35]. Merged reads were assigned to samples based on barcodes and truncated by removing the barcode and primer sequences. The DADA2 pipeline within QIIME2 was then applied as a quality control measure to remove chimeric sequences and filter any phiX reads present in marker gene Illumina sequence data. Quality plots of demultiplexed sequences revealed high quality scores of initial and distal base reads for a final sequence length of 250 bp. Sequence alignments were generated using the mafft plugin in QIIME2, and highly variable positions were masked. FastTree was used through the QIIME2 q2-phylogeny plugin to generate a phylogenetic tree from sequence alignments. Midpoint rooting was applied, and the resulting tree was used in downstream phylogenetic diversity analyses. Reads were rarefied to 12,932 sequences per sample and alpha diversity was determined with Faith’s Phylogenetic Diversity, Shannon Diversity, number of observed taxa (richness), and Pielou’s Evenness. Kruskal–Wallis pairwise comparisons were made to evaluate differences in community diversity. Weighted UniFrac measures were applied according to [36] to measure beta diversity. Taxonomic composition of the samples was determined using a Naive Bayes classifier trained on the Greengenes 13_8 99% OTUs according to QIIME2 to generate taxa tables and plots. Mean relative taxon abundance was calculated and compared between sites by merging metadata with QIIME2 taxon and feature tables in RStudio version (2023.12.0) using the qiime2R library. Phyla that made up less than 4% of the overall community abundance were collapsed into a category named “other.” Stacked bar charts were generated using ggplot2 for Rstudio. Nonmetric multidimensional scaling (NMDS) was performed in RStudio using vegan by importing weighted UniFrac distance matrices and running the NMDS on the distance matrix in two dimensions. Stress values were determined, and coordinates were merged with metadata. Environmental variables were fit to the NMDS plot using 999 permutations. Only significant environmental variables (*p* < 0.05) were extracted and fit to plot. Faith’s Phylogenetic Distance, Shannon Diversity Index, Pielou’s Evenness, and species richness vector files were imported into RStudio, and diversity indices were displayed in box plots using Rstudio qiime2R, vegan, and patchwork.

### 2.4. Metagenomic Library Preparation and Barcoding

Based on different pH and metals profiles, sites RB1, 9th St, and Dalla were further analyzed for ARGs and potential AMR. Metagenomic sequencing libraries were prepared using the Oxford Nanopore Technologies^®^ (ONT) Rapid Barcoding Kit V14 (SQK-RBK114.24) (Oxford, UK) according to manufacturer’s protocol. Briefly, 150 ng gDNA was prepared from each sample in a final volume of 10 μL of nuclease-free water. Genomic DNA was mixed with the respective kit supplied Rapid Barcodes and Fragmentation Mix. The mixture was incubated at 30° C for one minute and then 80° C for one minute to achieve DNA fragmentation and barcode-tag attachment. Samples were then pooled and ONT Rapid Adapter (RAP) was added. The final sequencing library was loaded onto a primed R10.4.1 flow cell (FLO-MIN114). Sequencing was performed on an Mk1C MinION portable sequencing device (ONT, Oxford, UK) for 72 h. Base-calling and demultiplexing to sort reads by respective barcodes was performed in real time using the MinKNOW software (6.0.14).

### 2.5. Antimicrobial Resistance Gene (ARG) Identification and Diversity Measures

Antimicrobial resistance genes were identified using the Fastq Antimicrobial Resistance workflow within EPI2ME (ONT, Oxford, UK). This workflow leverages the Resistance Gene Identifier (RGI) tool, which uses homology and SNP models to compare the sequencing reads directly against the Comprehensive Antibiotic Resistance Database (CARD) [37]. The number of ARGs detected per site was normalized for each gene based on the library size in average gene counts/mega base pair (mbp) according to [38]. Relative ARG abundance and antibiotic marker abundance for which each ARG corresponded were compared in RStudio using ggplot2. Alpha-diversity measures were calculated in RStudio using an ARG abundance matrix where rows represented individual sampling sites, and columns represented the normalized counts of each observed ARG. Species richness, Shannon Diversity Index, and Pielou’s Evenness were calculated for each sampling site using functions within the vegan package for Rstudio.

### 2.6. Relationships Between ARGs and Metals

To evaluate the relationships between metal concentrations and the abundance of ARGs across the three study sites (RB1, 9th St, and Dalla), a multivariate correlation analysis was performed in RStudio using libraries vegan and reshape2. Prior to analysis, the metal and ARG datasets were screened for zero variance. To prevent mathematical artifacts and undefined correlation coefficients, metals or ARGs that remained constant or showed non-substantive variation across the three sites were excluded from the analysis. Non-parametric Spearman’s rank correlation coefficients (ρ) were calculated to assess the strength and direction of the associations between the remaining metal and ARG profiles. Spearman’s method was selected due to its robustness in handling small sample sizes and ability to detect monotonic relationships without assuming normal distribution of the data. The resulting correlation matrix was visualized as a heatmap in RStudio using ggplot2.

## 3. Results

### 3.1. Site Characteristics

Iron was the predominant metal across all sites and reached a maximum of 478,000 ppm at RB1. This high concentration at the upstream sites was largely responsible for total metal concentrations. The sites with the highest total metal concentrations were RB1 (480,211 ppm), RB2 (447,237 ppm), and CC2 (426,542 ppm). In sharp contrast, Dalla (42,326.5 ppm), 9th St (42,782 ppm), and 32nd St (47,249.1 ppm) showed the lowest overall metal totals. The elemental breakdown revealed distinct patterns across the sites. Iron dominated, reaching a maximum of 478,000 ppm at RB1, where the highest concentration of Pb at 938 ppm was also detected. Aluminum was the second most abundant element, peaking significantly at CC3a with 138,000 ppm. In contrast, RB2 exhibited the maximum concentration of As at 181 ppm, while CC1 showed the highest Cu level of 143 ppm. The downstream sites, particularly Dalla and 9th St, registered the highest levels of Zn (835 ppm) and Mg (6940 ppm), respectively. Cadmium was the least abundant metal, often found below detectible limits (BDL) at RB1 and RB2, and peaking at a low 2.77 ppm at Dalla. Not surprisingly, the most striking difference in environmental conditions measured was that of pH. The BPMD sites ranged in pH from 3.0 to 3.8, except for RB2, where the pH was 8.0. Downstream sites were consistently neutral to slightly alkaline at 8.0–8.2. Dissolved oxygen concentrations varied somewhat with values generally higher in downstream sites with lower metal concentrations and higher pH. Temperatures downstream were considerably higher, which was expected given the 1360 m elevation loss between the RB1 site and Durango (Table 1).

### 3.2. Overview of the Illumina Sequencing Dataset

After filtering low-quality reads, chimeras, and spurious mitochondrial and chloroplast sequences, approximately 2,100,278 raw sequence reads of the 16S rRNA gene spanning the hyper variable V4 region were obtained from the 27 sediment samples. Read lengths averaged 250 bp and reads consisted of 24,046 Amplicon Sequence Variants (ASVs).

### 3.3. Taxonomic Profile and Abundance

**Figure 2:**
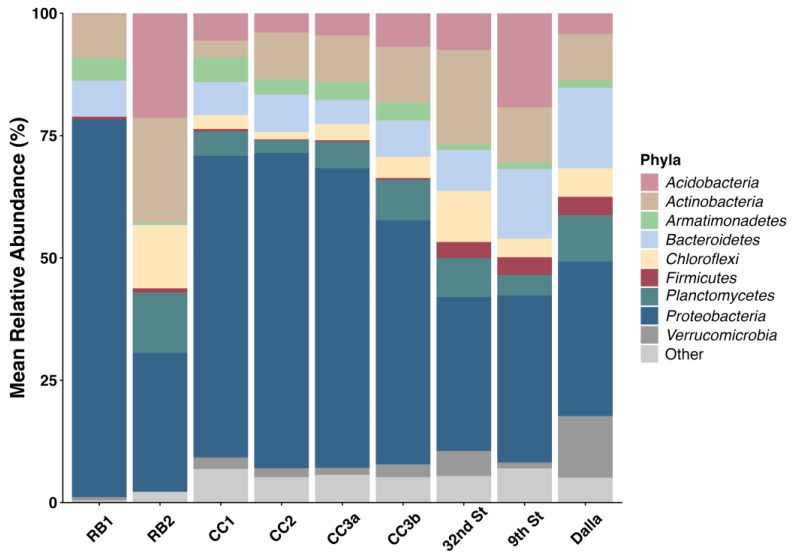
Relative abundance of bacterial phyla at each site. The “other” category consists of grouped phyla with <4% abundance. Sites are listed from upstream to downstream locations.

Altogether, 61 phyla were recovered; however, the general taxonomic pattern was mainly driven by differences in the abundance of seven major taxonomic groups. Most of these reads were affiliated with members of the Proteobacteria, followed by Acidobacteria, Actinobacteria, Bacteroidetes, Chloroflexi, Firmicutes, Planctomycetes, and to a lesser extent, Verrucomicrobia (Figure 2).

Proteobacteria dominated all sites but ranged from a mean of 19.7% at RB2 to 72.6% at RB1. The remaining BPMD sites ranged from 34.6 to 55.2% while downstream sites contained around 30% Proteobacteria. Greater taxonomic resolution revealed that within the Proteobacteria, bacterial classes differed considerably across sites. Alphaproteobacteria ranged from 10 to 13% in all sites except from RB1, where abundance dropped to 0.8%. Betaproteobacteria dominated the acidic sites, where mean abundance ranged from 20 to 40% then dropped to only around 7–10% in downstream neutral pH sites. Furthermore, Betaproteobacteria from the acidic sites consisted almost entirely of *Gallionella*, with the exception of RB1 where most of the Betaproteobacteria were unclassified. Gammaproteobacteria were most abundant from RB1 accounting for 30.7% but ranged from only 1.4% from RB2, to 10% from CC1 with all other sites falling within that range. Actinobacteria ranged from 2.9% from CC1 to 18% at 32nd St. Higher percentages of Actinobacteria were also detected from RB2 (14.6%) and 9th St (10.5%). Chlorflexi were most abundant from 32nd St (9.8%) and RB2 (8.6%) but abundance dropped to 3.5–5.4% at the remaining sites. Acidobacteria were commonly more abundant at the 9th St and RB2 sites and contributed to community similarities among the two sites with mean values at 17.4% to 14.6%, respectively. Acidobacteria abundance ranged from 3.4% to 7% from the remaining sites regardless of location.

The biggest taxonomic differences between the BPMD and the in-town sites were caused by Firmicutes, which were not detected in the BPMD sites, and Verrucomicrobiota, which were very rare in CC sites and absent in RB sites. Mean abundance in downstream sites was 1.6–4.5% and 4.8–11.8%, respectively. Greater taxonomic resolution revealed that overall, classified subtaxa in the neutral pH sites were Rhodobacteriales (*Paracoccus*), Saprospirales, Acidobacteria-6, and Acidobacteria RB41. The acidic sites, except for RB1, were dominated by Actinomycetales (*Microbacterium*) and Rhodospirallales (*Roseococcus*), while RB1 showed a unique profile and was dominated by unclassified Betaproteobacteria, Actinomycetales, Sphingobacteriales, and Fimbriimonadia. RB2 was the only site where Ktedonobacterales was abundant. The CC2, CC3, and 9th St sites shared Enterobacteriales *(Plesiomonas)* bacteria from the Gammaproteobacterial phylum. Meanwhile all three downstream sites harbored Burkholderiales family *Comamonadaceae.*

### 3.4. Community Beta Diversity

**Figure 3:**
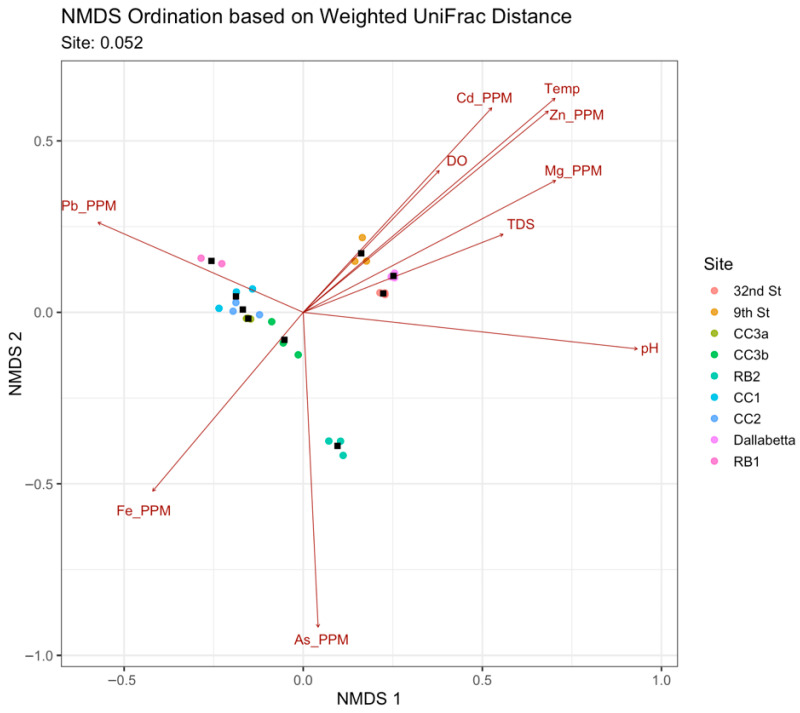
Nonmetric multidimensional scaling (NMDS) plot based on weighted UniFrac distance. The closer the sample points the more phylogenetically similar the microbial communities. Permanova *p*-value = 0.052 indicating significant effect size of the “site” factor on the observed community differences. Black squares depict the centroid of the three sample replicates for each site.

Nonmetric multidimensional scaling (NMDS) using the weighted UniFrac distance matrix generated in QIIME2 showed strong correlations between microbial community phylogenetic composition and environmental variables (Figure 3). The stress value of 0.052 suggested the plot was a highly reliable two-dimensional representation of community dissimilarities. Overall, microbial communities from sites in the BPMD were more alike than those from downstream sites in Durango. The longest arrow, pointing positively along NMDS 1 suggested that pH was the most significant environmental factor distinguishing the sites along the horizontal axis. Neutral pH sites downstream from BPMD, harbored distinct microbial communities from sites clustering on the left side of the plot, which came from acidic pH environments except for RB2 where the pH was 8.0. The NMDS 1 axis also suggested that differences in metal concentrations and composition played a role in structuring microbial communities. While Fe correlated strongly with the community structure found in RB1, CC1, CC2, CC3a, and CC3b, the RB1 microbial community is also highly correlated with Pb, differentiating RB1 microbial communities from others in the BPMD. Furthermore, NMDS axis 2 suggested that high As concentrations are a key factor differentiating the RB2 microbial community structure from other sites in the BPMD and from downstream sites with similar pH, making the microbial community from RB2 unique. Meanwhile, 32nd St, 9th St, and Dalla were associated with higher temperatures and DO, Cd, Zn, and Mg. Total dissolved solids (TDS) and neutral pH also played a role in structuring the communities from these sites. The gradient of increasing diversity downstream corresponded to a shift in environmental drivers, and the highest diversity sites 32nd St, 9th St, and Dalla being primarily influenced by general river characteristics such as TDS, Zn, and Mg, indicating a recovery from severe metal toxicity.

### 3.5. Community Alpha Diversity

**Figure 4:**
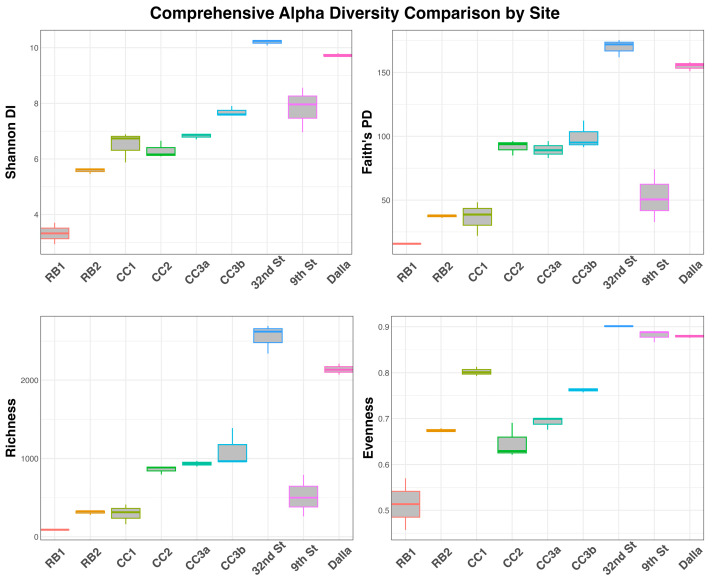
Comprehensive alpha diversity comparison by site. Box plots illustrating the microbial alpha diversity metrics across sampled sites (n = 3 replicates per site). Alpha diversity was calculated using four metrics: Shannon DI, Faith’s PD, community richness, and Pielou’s evenness. Sites are listed from upstream to downstream locations.

Alpha diversity metrics, including the Shannon Diversity Index (DI), Faith’s Phylogenetic Diversity (PD), and Species Richness, consistently exhibited a strong positive gradient across the sampling sites, culminating in the lowest values at RB1 and the highest at 32nd St. Species Richness showed the largest increase in magnitude, rising sharply from approximately 100 observed ASVs at RB1 to over 2000 at 32nd St, excluding the 9th St site. Differentiation in phylogenetic diversity was highly significant across groups (Faith’s PD, *p* = 0.003), with 32nd St being phylogenetically more diverse than all other sites (*p* = 0.04), save for a marginal difference with Dalla (*p* = 0.08). Notably, 9th St showed more similarity in phylogenetic diversity to RB2 and CC1 (*p* = 0.27 and 0.51, respectively), which was supported through observations of high Acidobacteria abundance among the sites, and the absence of Verrucomicrobia at 9th St (compared to the other downstream sites). Similar patterns emerged for the Shannon DI; however, when considering diversity without phylogenetic differences, 9th St became more similar to 32nd and Dalla, but still not significantly different from the CC and RB2 sites. In contrast, Pielou’s Evenness was notably low (0.5–0.6) at the RB1 site, signifying community dominance by a few taxa. As metal concentrations declined among downstream sites, evenness increased, suggesting a highly balanced distribution of taxon abundance as stream health improved. Statistically, the evenness metric showed significant differences across most pairwise comparisons (*p* = 0.04–0.05), though no significant differences were found between Dalla, 9th, and 32nd St (*p* = 0.51) (Figure 4).

### 3.6. ARG Profile and Abundance

**Figure 5:**
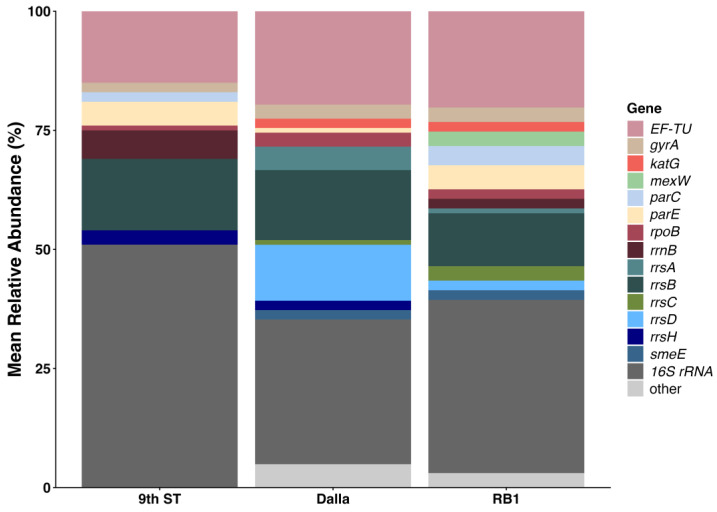
Relative abundance of ARGs detected by CARD query. Those appearing at <1% frequency were collapsed into the other category.

**Figure 6:**
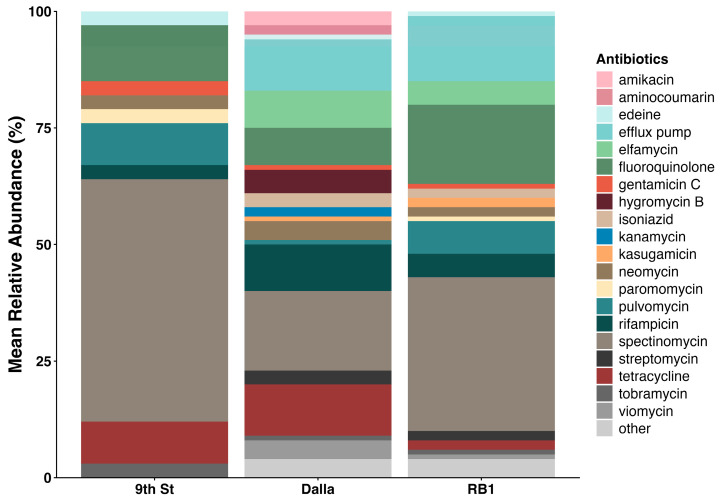
Relative abundance of antibiotic markers encoded for by ARGs detected by CARD query. Those appearing at <1% frequency were collapsed into the other category.

The three sites surveyed for ARGs revealed a total of 31 distinct genes in the CARD query yielding 70% or higher identity with genes that carry mutations leading to antibiotic resistance or encode for efflux pumps. Among them, genes encoding for efflux pumps were most prevalent at 48% (15/31), followed by aminoglycosides 26% (8/31), fluoroquinolones 13% (4/31), and tetracyclines 9.7% (3/31) (Table 2). And, while similarities in resistance profiles were detected, the number of genes that encoded for resistance to specific antibiotics varied. Among the collection sites, RB1 revealed 15 ARGs associated with efflux pumps and 16 ARGs associated with mutations that lead to resistance of 13 antibiotics (AMR markers). Eight ARGs associated with eight AMR markers were found in 9th St sediments; however, none associated with efflux pumps were detected. Dalla yielded 6 ARGs associated with efflux pumps and 15 genes associated with 11 AMR markers (Table 2, Figure 5 and Figure 6).

### 3.7. AMR Marker Diversity

The relative abundance of resistance markers for various antibiotics across the three locations, 9th St, Dalla, and RB1, differed; however, the Dalla and RB1 profiles were more alike than that of 9th St (Figure 5). Overall, spectinomycin markers were the most abundant across all sites, accounting for up to 50% of the total relative abundance. The 9th St site was dominated heavily by spectinomycin, fluoroquinolone, and tetracycline markers. Meanwhile, Dalla and RB1 featured the presence of efflux pump mechanisms. Dalla and RB1 differed in that kanamycin, amikacin, aminocoumarin, and hygromycin B markers were present in the Dalla site. Meanwhile a paromomycin marker was detected solely in the RB1 site. Common resistance markers included fluoroquinolone, rifampicin, and pulvomycin, which were present at varying levels in all three locations.

Across all three locations, the 16S rRNA gene marker was the most abundant, though its proportion varied. It occupied roughly 50% of the relative abundance in the 9th St sample but dropped to approximately 35–40% in the Dalla and RB1 samples. The *EF-Tu* gene, which is involved in protein synthesis, consistently represented the second-largest category, making up about 15–20% of each sample. Additional ribosomal RNA gene marker diversity was detected through various *rrs* genes (*rrsA*, *rrsB*, *rrsD*, *rrsH*). Notably, the Dalla sample had a higher relative abundance of *rrsD* compared to the other two sites. Lower but consistent ARG abundance was composed of *gyrA*, *parC*, *and parE*, which are associated with DNA replication and fluoroquinolone resistance. The *rpoB* gene involved in RNA transcription was most abundant in the 9th St site. Genes with relative abundance of < 1% were collapsed into the “other” category. The Dalla and RB1 samples contained a visible other category, which was negligible in the 9th St sample. It is noteworthy, however, that all collapsed genes for the RB1 site encode for efflux pumps and were various *mex* and *sme* genes (Table 2). The Shannon DI, Pielou’s evenness, and richness measures applied to normalized ARG counts suggested that RB1 had slightly higher ARG diversity than Dalla while 9th St was substantially less diverse. As a result, site RB1 had higher numbers of ARGs when compared to the other sites, as indicated by a richness value of 27, compared to 21 at Dalla, and only 9 at 9th St. However, Pielou’s Evenness suggested that all sites had similar gene distributions (Table 3).

### 3.8. Correlation Between Metals and ARGs

**Figure 7:**
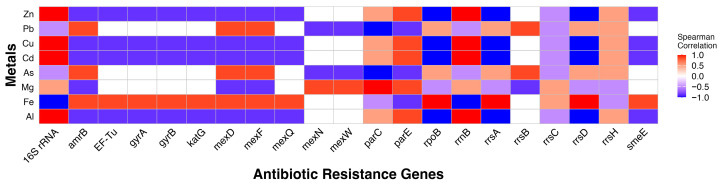
Spearman’s Rank Correlation Analysis of metal concentrations and ARG abundances. The heatmap illustrates the strength and direction of associations between metal concentrations and the relative abundance of resistance determinants across three sampling sites: RB1, 9th St, and Dalla. The color gradient represents Spearman’s rank correlation coefficient (ρ) ranging from −1.0 (dark blue; perfect negative correlation) to 1.0 (red; perfect positive correlation). Tiles in red signify potential co-selection hotspots, where increasing metal concentrations are perfectly correlated with high abundance of specific ARGs.

Spearman’s correlation analysis identified a robust co-occurrence pattern between metal concentrations and specific ARGs (Figure 7). Most notably, 16S rRNA, *parE*, and *parC* abundance exhibited perfect (ρ = 1.0) to high (ρ = 0.85) positive correlations with Zn, Cu, Cd, and Al, suggesting that these metals may act as primary drivers for the selection of the target-site mutations in 16S rRNA, *parE*, and *parC* identified via the CARD algorithm. Furthermore, genes encoding for multidrug efflux pumps, *mexD*, *mexF*, and *mexQ* showed perfect positive correlations with Fe, Pb, and As. This suggested that these metals increased the microbial community’s “pump-based” resistance to multiple antibiotics. Meanwhile, Fe was negatively correlated with 16S rRNA and positively correlated with *amrB*, *EF-Tu*, and the *gyrA/gyrB* complex. Aluminum and Cu are negatively correlated with most of the efflux and DNA-modifying genes (*gyrA*, *gyrB*, *katG*, *mexD*, *mexF*, *mexQ*) (Figure 7).

## 4. Discussion

The study herein aimed to characterize microbial communities among river sediments collected from the BPMD, an area characterized by legacy mining, resultant heavy metal contamination, and low pH conditions as compared to downstream sites lacking mining impacts. Additionally, three sites were surveyed for ARGs and AMR markers to determine diversity and abundance among sites with differing geochemical characteristics and microbial diversity. Many studies have shown that metals and pH can select for specific microbial communities and reduce community diversity [19,20,21,39,40]. Therefore, it was not surprising that the microbial landscape of our study area was sharply divided by pH and metal toxicity. Sites influenced by high Fe, Pb, and As concentrations and low pH consistently exhibited the lowest diversity and evenness and were dominated by Gamma- and Beta-proteobacteria. However, differences were noted among the BPMD sites depending on metal concentrations and relative metals proportions. And, despite some BPMD sites being geographically proximal to each other (RB1 and 2 and CC3a and CC3b), pH and metals heterogeny was discovered. Site RB1 had the highest overall metal concentration but a higher proportion of Pb than other sites. As a result, microbial communities were less diverse, showed elevated abundance of unclassified Betaproteobacteria, lacked Gallionelles, and instead were characterized by Actinomycetales, and on a more resolute taxonomic level, Sphingobacteriales and Fimbriimonadia. Studies have shown that many Betaproteobacteria and Actinomycetales are active in Pb sequestration and efflux possessing *pbr* operons, which allow them to actively pump Pb(II) ions out of the cell or sequester them within the cell wall to prevent metabolic interference [41]. Meanwhile, Sphingobacteriales and Fimbriimonadia contribute to biofilm formation through extracellular polymeric substance (EPS) production, which acts as a physical barrier, binding lead outside the cell membrane and reducing its bioavailability. The CC sites, where Fe had the most influence and Pb concentrations were proportionally lower, were characterized by Gallionellales and Acidobacteriales, taxa specialized in iron transformations and a hallmark of AMD impacts [42,43]. *Gallionella* are known iron-oxidizing bacteria that thrive at the oxic–anoxic interface gaining energy through iron oxidation, while often forming twisted stalks of iron oxyhydroxides [43]. Acidobacteriales are associated with both the oxidation and dissolution of Fe and Cu, playing critical roles in the biogeochemical cycling of metals under low pH conditions [44]. A neutral pH (8.0) like downstream sites, Fe concentrations similar to other BPMD sites, and relatively high As concentrations acting as the primary selective pressure, made RB2 ecologically unique. Taxonomy shifted to a community dominated by Actinobacteria, Acidobacteria, and Chloroflexi, specifically Ktedonobacterales. The unique presence of Ktedonobacterales suggested a highly specialized niche at RB2. These microbes often facilitate arsenic transformation (arsenite oxidation or arsenate reduction) to manage toxicity [41]. Downstream as the pH neutralized (~8.0), and Zn and Cd concentrations rose, phylogenetic diversity reached its peak. Firmicutes and Verrucomicrobiota became increasingly abundant members of the downstream communities and Proteobacterial abundance decreased. Firmicutes can be found across many environments and are a common bacterial phylum found in urban streams, often serving as indicators of organic pollution, including sewage and animal waste [45]. Verrucomicrobiota, typically found in more stable, nutrient-rich environments, indicated a transition away from the extreme stress of the upstream mining sites. Downstream sites also harbored members of the *Comamonadaceae*, which are known to correlate strongly with several anthropogenic nutrients including nitrates, are generally ubiquitous, and have previously been associated with urban streams [39]. The presence of Chloroflexi and Actinobacteriota may serve as biological indicators for Zn, as these groups often possess high tolerance and specific resistance genes for Zn rich sediments [46]. These findings supported water quality associated with urban areas and were not surprising given the site proximity to agricultural inputs, stormwater and surface water runoff, and high recreational use of the Animas River.

The distribution and diversity of ARGs and AMR markers across the surveyed sites revealed a complex landscape of resistance, likely driven by a combination of heavy metal co-selection and urban anthropogenic influences. Our findings indicated that while certain housekeeping genes capable of inferring AMR through point mutations, like 16S rRNA and *EF-Tu*, remained relatively stable across sites, the specific ARG profiles differentiated the metal impacted site RB1, from downstream sites 9th St and Dalla. The most striking feature of the ARG profiles was the dominance of efflux pump mechanisms, which accounted for 48% (15/31) of the distinct genes identified. Additionally, correlation analysis suggested that high concentrations of Fe and Pb as found in the RB1 site, may act as co-selective factors for efflux pump genes (Figure 7). The high richness (27 ARGs) at RB1 indicated a specialized community where survival depended on a broad array of resistance strategies and mainly a diversity of efflux pump mechanisms. Efflux pumps, specifically the *mex* and *sme* systems identified at RB1, are a common bacterial response to environmental stress, particularly metals. Because efflux pumps are often non-specific, meaning they can expel both antibiotics and heavy metals from the cell, they are one of the premier examples of metal and antibiotic resistance co-selection through cross-resistance [4,14,37], and can result in multidrug resistance. For example, in *Pseudomonas aeruginosa*, one of the main pathogens associated with respiratory tract and healthcare-associated infections, the *mex* *AB, XY, CD*, and *EF* efflux pumps have been associated with antibiotic resistance [32]. The same that were detected primarily from RB1 and secondarily from Dalla. The presence of these genes mainly in RB1 and high to perfect correlations to Pb, Fe, and As, suggested a selective pressure at RB1 that maintains ARG presence through efflux pump co-resistance [4,14]. While 9th St had the highest relative abundance of 16S rRNA (~50%) possessing mutations that can inhibit the binding of bacteriostatic antibiotics, the selective pressures required to maintain a diverse “resistome” appeared less intense at 9th St than those found at the upstream mining-impacted site. Higher concentrations of Zn, Cu, Cd, and Al tended to favor ARGs subject to target site mutations or alterations such as ribosomal genes, *parC*, and *parE*, rather than efflux pump systems.

Similarities between the Dalla and RB1 profiles including similar ARG and AMR marker diversity and abundance suggested that mining-impacted and downstream sites may share a common “core resistome” characterized by efflux-mediated multidrug resistance. The stark contrast at 9th St, which lacked efflux pumps and showed significantly lower richness, may have been the result of sampling bias, or a differential influence of metals or anthropogenic inputs that were not specifically measured.

The prevalence of spectinomycin markers across all sites, up to 50% relative abundance, suggested a baseline level of aminoglycoside resistance in these aquatic environments. However, the differentiation of “minority” genes (the <1% “other” category) provided insight into site-specific stressors. For example, the detection of paromomycin markers was also unique to RB1, alongside its rich profile of efflux pumps, which again underscores its status as an extreme environment. Dalla displayed a unique signature of aminoglycosides (kanamycin, amikacin, hygromycin B, and aminocoumarins). This increased variety in antibiotic markers, despite lower overall ARG richness compared to RB1. Consistent alignment during metagenomic analysis to mutations in the *gyrA*, *parC*, and *parE* across sites confirmed that resistance to synthetic antibiotics like fluoroquinolones is widespread, potentially integrated into the stable chromosomal makeup of the resident microbial communities [47]. Although RB1 showed higher diversity in efflux pump genes when compared to downstream sites, the overall ARG diversity of RB1 and Dalla were similar despite the microbial diversity of Dalla being significantly higher than that of RB1. Our results therefore were not consistent with findings that more diverse communities lead to lower abundance and diversity of ARGs. Rather, ARG and AMR markers were unique for each site based on metal concentrations. Zinc, Cu, Cd, and Al appeared to form a “co-selection cluster” that specifically targeted the ribosome (16S rRNA) and topoisomerases (*parE*), while Pb, As, and Fe correlated with active transport through efflux pumps like those encoded by *mex* genes, *spe* genes, and *amrB*. Iron also appeared to co-select for *EF-Tu*, *gyrA*, and *gyrB* suggesting that Fe is an important metal for co-selection of antibiotic target alteration. However, it should be noted that our sample sizes were small, and we were unable to employ qPCR or other quantitative methods for this study, which emphasizes resource limitations in rural areas. Despite this, the ARG profile in RB1 was unique compared to other sites and was dominated by efflux pump systems that correlated to the metals found therein, a hallmark of metal resistance and co-selective pressures. This is an important finding as multidrug resistance becomes a growing clinical problem, and environmental contamination continues to encroach on poorly resourced communities. Although sampling efforts were resource limited for this study, we provided insights into potential AMR stemming from mining impacted areas into rural downstream communities. Antibiotic resistance is a growing public health issue and rural clinical settings like those in southwestern CO disproportionately lack antibiotic stewardship programs and antibiotic resistance tracking relative to urban counterparts. Our study raises awareness of the importance of providing antimicrobial tracking techniques to resource limited communities and could help provide information needed for better antibiotic use recommendations or environmental monitoring. A standardized ARG surveillance system and an environmental focus on AMR distribution in addition to clinical surveillance programs could add a valuable aspect of AMR prevention in the future.

## Figures and Tables

**Table 1:** Concentration of metals and environmental conditions in river sediments at each collection site. Parts per million (ppm) are equivalent to mg/kg. BDL, below detectable limit. DO*, dissolved oxygen; TDS**, total dissolved solids.

Site	Al ppm	As ppm	Cd ppm	Cu ppm	Fe ppm	Mg ppm	Pb ppm	Zn ppm	pH	DO* (mg/L)	Temp °C	TDS**
RB1	847	24	BDL	8.99	478,000	219	938	174	3.0	9.7	5.5	521.6
RB2	7260	181	BDL	57.6	437,000	2450	168	120	8.0	10.3	6.1	457.5
CC1	12,700	28.4	1.93	143	108,000	4310	404	393	3.8	12.7	12.7	622.7
CC2	4870	23.8	0.654	42.5	420,000	917	331	357	3.8	12.4	7.5	333.8
CC3a	138,000	5.79	0.691	14.5	17,600	3770	38.1	78.9	3.6	8.4	8.0	457.5
CC3b	10,100	84.2	1.05	68.8	249,000	4130	217	304	3.6	8.5	8.0	457.6
32nd St	11,300	9.14	2.01	68.9	28,700	6270	147	752	7.7	12.4	17.3	487.5
9th St	10,900	7.96	1.71	47.3	24,200	6940	117	568	8.0	13.0	19.8	500.5
Dalla	11,700	8.74	2.77	57	23,900	5690	133	835	8.2	11.2	20.9	481

**Table 2:** ARGs and the antibiotics for which they encode resistance; antibiotic classes to which each ARG and marker belongs. Point mutations within ribosomal genes (16S rRNA, *rrn*, *rrs*, *EF-Tu. rpo*) and housekeeping genes (*par*, *gyr*, *ala*) can cause antibiotic binding site alterations which render bacteria antibiotic resistant. Genes encoding for efflux pumps enable elimination of antibiotics from the cell.

Antibiotic Class	AMR Marker	Gene(s)
aminoglycosides	amikacin	16S rRNA
	gentamicin	16S rRNA
	hygromycin B	*rrsA*, *rrsB*
	kanamycin A	*rrsA*, *rrsB*
	kasugamycin	*rrsC*
	spectinotmycin	16S rRNA, *rrnB*, *rrsB*, *rrsD*, *rrsH*, *rpsL*
	streptomycin	16SrRNA, *rrnB*, *rrsB*
	tobramycin	16S rRNA, *rrsB*
	neomycin	16S rRNA, *rrsA*, *rrsB*
	paromomycin	*rrsB*
antimycobacterials	isoniazid	*katG*
efflux pumps	efflux pumps	*amrB*, *ceoB*, *mdtB*, *mexB*, *mexD*, *mexF*, *mexI*, *mexK*, *mexN*, *mexN*, *mexQ*, *mexY*, *mfd*, *smeB*, *smeE*
elfamycins	elfamycin	*EF-Tu*
	kirromycin	*EF-Tu*
	pulvomycin	*EF-Tu*
fluoroquinolones	fluoroquinolones	*gyrB*, *gyrA*, *parC*, *parE*
gyrase inhibitor	aminocourmarin	*alaS*, *gyrB*, *parY*
peptide	edeine	16S rRNA
rifamycin	rifampicin	*rpoB*
tetracyclines	tetracycline	16S rRNA, *rrnB*, *rrsB*
tuberactinomycins	viomycin	*rrsB*

**Table 3:** Alpha diversity measures ARGs by site.

Site	Shannon DI	Pielou’s Evenness	Richness
9th St	1.5	0.7	9
Dalla	2.1	0.7	21
RB1	2.2	0.6	27

## Data Availability

The original contributions presented in the study are included in the article, further inquiries can be directed to the corresponding author.
